# Characterization of multiple insecticide resistance in *Anopheles gambiae* (Diptera: Culicidae) from Pointe Noire, Republic of the Congo

**DOI:** 10.1186/1475-2875-9-S2-P17

**Published:** 2010-10-20

**Authors:** Lizette L Koekemoer, Belinda S Spillings, Riann Christian, Oliver S Wood, Maria Kaiser, Ryan Norton, Kwang S Choi, Basil D Brooke, Richard H Hunt, Maureen Coetzee

**Affiliations:** 1 Vector Control Reference Unit, National Institute for Communicable Diseases, NHLS, Private Bag X4, Sandringham, 2131, South Africa; 2 Malaria Entomology Research Unit, School of Pathology, University of the Witwatersrand, South Africa; 3 School of Animal, Plant and Environmental Sciences, University of Witwatersrand, Johannesburg, South Africa

## Background

An integrated malaria vector control programme utilises three main interventions: vector control, accurate clinical diagnosis and treatment. Initiation of a vector control programme requires baseline information on vector species composition, infectivity rates of the target vector populations and susceptibilities of target vector populations to insecticides. The baseline information needed for malaria vector control is still limited in some countries, including the Republic of the Congo, and the aim of this study was to obtain this information from Pointe Noire on the Congo coast.

## Materials and methods

Field sampling was conducted during April 2009 in the village of Kouilou Potash, close to the city of Pointe Noire. *Anopheles gambiae s.l* mosquitoes were collected resting indoors. Samples were used for the following analyses: standard WHO insecticide susceptibility testing, species identification and *Plasmodium* sporozoite detection. The following assays were conducted to characterize resistance mechanisms: synergist analysis, biochemical analysis, microarray analysis and hydrolysis probe/TaqMan® and Restriction Fragment Length Polymorphism (RFLP) analysis for target site resistance associated mutations.

## Results

The only malaria vector species collected was *An. gambiae* S form with a high *P. falciparum* infection rate (9.6%). Multiple insecticide resistance was detected in this population with full susceptibility to only one insecticide class, the organophosphates. Dieldrin and DDT resistance was attributed to target site resistance (the *rdl* and L1 014F/ L1014S *kdr* mutations respectively) while pyrethroid resistance was mainly attributed to monooxygenase mediated detoxification.

## Conclusion

The role of various insecticide resistance mechanisms revealed a complex association between metabolic detoxification and reduced target site sensitivity. The high level of DDT resistance in Pointe Noire clearly negates use of this insecticide for malaria vector control in the region. As only pyrethroids are currently used for the treatment of bed nets, it would be advantageous in terms of resistance management to use either organophosphates or carbamates for indoor residual house spraying. Further, the geographical extent of the multiple insecticide resistance observed in this study should be investigated.

